# Cost evaluation of an exercise oncology intervention: The exercise in all chemotherapy trial

**DOI:** 10.1002/cnr2.1490

**Published:** 2021-07-08

**Authors:** Melanie Potiaumpai, Shawna E. Doerksen, Vernon M. Chinchilli, Hongke Wu, Li Wang, Rachel Lintz, Kathryn H. Schmitz

**Affiliations:** ^1^ Public Health Sciences, Penn State College of Medicine Hershey Pennsylvania USA

**Keywords:** cancer care delivery, cost–benefit analysis, exercise oncology, healthcare utilization, supportive care

## Abstract

**Background:**

There is strong evidence supporting the efficacy of exercise oncology programs to improve physical and psychosocial outcomes during active treatment. However, there is a paucity of evidence on the effect of exercise on healthcare utilization and cost analyzes of exercise oncology programs.

**Aims:**

Our objective was to assess the effects of a pragmatic exercise oncology program (ENACT) during active chemotherapy treatment on healthcare utilization and associated costs.

**Methods:**

We conducted post‐hoc analyzes on 160 ENACT participants and 75 comparison participants matched on cancer site, stage, age range, and gender. We obtained complete healthcare utilization histories for each patient (specific to emergency department [ED] visits and hospital admissions) coinciding with their participation in ENACT. A sub‐analysis was conducted for advanced stage breast, gastrointestinal, and pancreatic cancer patients.

**Results:**

Healthcare costs for patients who participated in the ENACT exercise oncology intervention were numerically lower than healthcare costs for the comparison group, even after accounting for the cost of the intervention. However, the differences were not statistically significant.

**Conclusion:**

Our findings suggest that an exercise oncology program during active chemotherapy treatment are at least cost neutral for all cancer patients, including advanced stage cancers. Additional research is warranted to evaluate the potential for exercise oncology programs to reduce healthcare utilization, particularly in advanced cancer patients.

## 1 INTRODUCTION

Patients who receive chemotherapy average two emergency department (ED) visits and one hospitalization per year compared to the national average of 0.17 ED visits and 0.055 hospitalizations among the general population.^1^ Fifty percent of the ED and hospitalization visits in patients who receive chemotherapy are potentially avoidable^2^, ^3^, ^4^ and pose a high morbidity and mortality risk.^5^ Reducing avoidable ED visits and hospitalizations has implications in: improving the financial health of health systems that provide cancer care, calculating hospital performance, and lessening the economic burden for patients, payers, and society.^6^ Previous supportive care interventions that have attempted to address this pressing healthcare utilization issue have utilized more costly approaches such as additional nursing time, and/or advanced monitoring systems.^6^, ^7^, ^8^

Over 680 randomized controlled trials have clearly demonstrated that exercise is beneficial and effective for numerous health‐related outcomes during chemotherapy for multiple cancer diagnoses.^9^, ^10^ These include fatigue, physical function, quality of life, anxiety, depression, sleep, and breast‐cancer related lymphedema.^10^ Despite the extensive body of scientific evidence, only 30%–47% of cancer survivors are adequately meeting exercise guidelines.^11^, ^12^ Further, there is no similar body of scientific evidence that provides data on the proportion of patients who are adequately active during chemotherapy. Contributing to the low number of exercising patients is the low proportion of exercise referrals made by health care providers during infusion therapy: it is estimated that 9% of nurses and up to 23% of physicians refer their patients on active cancer therapy to exercise programs.^12^, ^13^, ^14^, ^15^ A major barrier to referral and implementation to exercise oncology programs is cost.^9^ Exercise oncology programs are not presently covered by third party payers in the United States and are seen as an unrecoverable cost on the part of patients, payers, and society.

The perception of exercise oncology programs as an expensive burden could potentially be eliminated if it could be demonstrated that exercise programming can reduce healthcare utilization (i.e., ED visits, hospitalizations) during chemotherapy. Even if the costs of an intervention were neutral compared to the elevated costs of healthcare utilization, this could be of interest to providers, health systems, and payers. To address this pressing issue, our team used data from a pragmatic pilot trial, Exercise iN All ChemoTherapy (ENACT). The ENACT trial demonstrated that embedding a cancer exercise trainer into the chemotherapy infusion suite to counsel and prescribe home‐based resistance and aerobic exercise is highly acceptable to healthcare providers and patients, and is feasible for patients to complete.^16^ For this post‐hoc analysis, we retrospectively examined healthcare utilization within trial participants and matched comparisons, to discern if exercise might alter these potentially avoidable costs.

## 2 METHODS

The ENACT trial was a mixed‐methods pre‐post single group pragmatic trial to assess the feasibility and acceptability of embedding an exercise trainer into the chemotherapy infusion suite from the perspective of clinicians and patients at the Penn State Cancer Institute (PSCI) (NCT03461471). For this post‐hoc analysis of the trial, we further identified gender‐, age‐, and cancer site‐ and stage‐matched patients through the electronic medical record. The Penn State Human Subjects Protection Office approved this protocol and all patients provided written consent prior to any study‐related activities.

### 2.1 Patient description

To be eligible for participation, patients had to be seen at PSCI for outpatient cancer infusion therapy between April 2017 and October 2018, be 18 years of age or older, have an Eastern Cooperative Oncology Group (ECOG) performance status of 0–2,^17^ and be receiving infusion therapy for a solid tumor, regardless of stage of cancer. Patients were excluded if they were pregnant, if there was evidence in the medical record of an absolute contraindication for exercise based on the American Heart Association guidelines,^18^ or if the medical oncologist or exercise trainer identified a diagnosis that would make unsupervised exercise unsafe.

### 2.2 Comparison group

Patients who served as the comparison group were selected from the ENACT patient directory of approached patients who were either not interested in participating or were excluded for nonmedical reasons such as timing (e.g., identified as they were nearing completion of cancer infusion therapy). Therefore, patients in the comparison control group shared identical inclusion and exclusion criteria as patients in ENACT. Further, for the sub analysis of advanced breast, gastrointestinal (GI), and pancreatic cancer ENACT patients, comparison group patients were matched based on cancer site, stage, age range, and gender.

We extracted ED visits and hospitalizations from the electronic medical record for all participants. We recorded information on ED visits and inpatient hospitalization stays based on the start date of when the patient was approached for participation in ENACT until the end of their primary treatment for nonmetastatic patients or 6 months later for metastatic patients, coinciding with study duration of ENACT patients. The length of time for comparison patients was matched.

### 2.3 Exercise intervention

The exercise intervention has been described previously.^16^ Briefly, the home‐based exercise intervention consisted of a mix of five elements: aerobic, resistance, balance, flexibility, and rest.

The resistance training prescription contained specific frequency, intensity, and time for each exercise. Although resistance training was the main exercise prescription for the majority of patients, aerobic exercise was included if it was possible, based on the functional capacity of the patient. To track completion of the exercises, each patient was provided personalized exercise logs along with an exercise manual that contained visual instructions on safe and proper exercise form and technique. Patients met with the exercise trainer at each infusion visit to review any exercise‐related symptoms or injuries, modify the exercise prescription if necessary, and receive new exercise logs.

### 2.4 Measurements

To identify ED visits and hospitalizations, staff reviewed all encounter notes from the start date (ENACT intervention start date or a matched comparison start date) and forward by 6 months or to the end of infusion therapy (if less than 6 months). Reports of ED visits and hospitalizations at all locations (Penn State and other health systems) were treated the same.

ED costs were calculated using the average cost of $1389.00 per ED visit.^19^ Hospitalization costs were calculated using the average cost of $10 700.00 per night of hospitalization admission.^20^ Intervention costs were estimated by evaluating the cost of staff time for screening patients in the medical record, the interventionist time per patient, multiplied by hourly wage including fringe, and equipment cost. We have previously reported this intervention cost.^16^ Overall total cost from the payers' perspective were calculated by summing up the costs of the ED visits, hospitalizations, and the intervention.

### 2.5 Statistical analysis

Descriptive statistics were used to report participant characteristics. The *t* tests and chi‐square tests were used to compare the intervention and comparison group patients, as appropriate. Because the cost data display right skewness and contain numerous zero values, we assumed a zero‐inflated lognormal distribution for the cost data. We derived maximum likelihood estimates of the parameters (mean and variance on the natural logarithmic scale, and the probability of a zero cost) for each of the ENACT and comparison groups. We then estimated the overall geometric mean for each group as the probability of a nonzero cost times the exponentiation of the mean on the logarithmic scale. Finally, we compared the geometric means via an approximate *t* test. All analyzes were adjusted for number of comorbidities. We used PROC NLMIXED of SAS, Version 9.4, for these calculations. Both arithmetic and geometric means are presented.

## 3 RESULTS

The characteristics of the study participants are described in Table 1. They ranged from 30 to 82 years, and on average were 60 years of age. There were slightly more women than men and 94% self‐reported white race. The most common tumor types included in the study were breast, GI, and pancreatic. Forty four percent of the consented patients had stage 4 cancer. Patients in ENACT had an average of 1.18 (SD = 1.29) comorbidities at baseline.

**TABLE 1:** Demographic and clinicopathological features of consented patients

Characteristic	Consented (*n* = 160)	Concurrent comparisons (*n* = 75)	*p* value	Stage 3 and 4 breast, GI, pancreatic in ENACT (*N* = 71)	Stage 3 and 4 breast, GI, pancreatic concurrent comparisons (*n* = 37)	*p* value
Age (years ± SD)	59.80 ± 11.42	60.69 ± 9.89	.57	58.90 ± 11.20	60.46 ± 11.00	.57
Gender (N [%])						
Female	98 (61.3)	42 (56.0)	.44	48 (67.6)	24 (64.9)	.78
Male	62 (38.7)	33 (40.0)		23 (32.4)	13 (35.1)	
Race (N [%])						
White	151 (94.38)	69 (92.00)	.49	67 (94.37)	32 (86.49)	.33
Black or African American	7 (4.38)	2 (2.67)		3 (4.23)	2 (5.40)	
Others	2 (1.25)	4 (5.33)		1 (1.40)	3 (8.11)	
Tumor type (N [%])						
Breast	35 (21.88)	17 (22.67)	.73	15 (21.13)	9 (24.32)	.96
GI	48 (30.00)	25 (33.33)		40 (56.34)	20 (54.06)	
Pancreatic	22 (13.75)	11 (14.67)		16 (22.53)	8 (21.62)	
Melanoma	12 (7.50)	6 (8.00)				
Other	43 (26.90)	16 (21.33)				
Tumor stage (N [%])						
1	13 (8.13)	3 (4.00)	.12			
2	29 (18.13)	15 (20.00)				
3	37 (23.13)	25 (33.33)		28 (39.44)	18 (48.65)	.67
4	71 (44.38)	31 (41.33)		39 (54.93)	18 (48.65)	
Unknown	10 (6.25)	1 (1.34)		4 (5.63)	1 (2.70)	
# of Existing comorbidities	1.18 ± 1.29	1.77 ± 1.54	.002	1.29 ± 1.39	1.73 ± 1.54	.14
0	64 (40.0)	19 (25.3)	.03	25 (35.2)	9 (24.3)	.25
1–2	74 (46.3)	32 (42.7)	.61	36 (50.7)	17 (45.9)	.64
3+	22 (13.8)	24 (32.0)	.001	10 (14.1)	11 (29.7)	.06
Pain (vital sign at baseline)	0.91 ± 1.87	1.01 ± 2.04	.71	0.85 ± 1.80	1.14 ± 1.81	.43
ECOG						
0	73 (45.6)	29 (38.7)	.32	35 (49.3)	12 (32.4)	.09
1	26 (16.2)	17 (22.7)	.24	18 (25.4)	10 (27.0)	.93
2	3 (1.9)	5 (6.7)	.06	1 (1.4)	3 (8.1)	.08
Missing	58 (36.3)	24 (32.0)	.46	17 (23.9)	12 (32.4)	.41

Also in Table 1, we provide the same demographic and clinical description of the 75 participants who served as concurrent comparisons. They shared a similar average age of 60 years, a slightly higher proportion of women to men, 92% self‐reported white race, common tumor types of breast, GI, and pancreatic cancer. Forty one percent of the matched comparisons had stage 4 cancer. Matched comparison patients had an average of 1.77 (SD = 1.54) comorbidities at the time of baseline. There was a statistically significant difference in the number of existing comorbidities at baseline between all ENACT and matched comparison patients (*p* = .002). Pain, as reported at baseline, did not differ between ENACT patients and matched comparisons (*p* = .71). ECOG physical status ratings ranged from 0–2, with ECOG scores of 0 or 1 assigned to over 60% of patients for both ENACT and matched comparisons.

ENACT patients of the sub analysis of advanced (Stage 3 and 4) breast, GI, and pancreatic cancer patients were on average 59 years of age, were slightly more women than men, were majority of white race (94%), 21% breast cancer, 57% GI cancer, and 22% pancreatic cancer. The majority of advanced stage patients in ENACT were Stage 4 (55%), followed by Stage 3 (39%) and 5% of patients did not have clear a staging report available; however, physicians confirmed the patients' metastatic state. Advanced stage patients in ENACT had an average of 1.29 (SD = 1.39) comorbidities at the time of baseline. Patients in the concurrent comparison group of this sub analysis of advanced breast, GI, and pancreatic cancer patients shared a similar average of 59 years of age, higher proportion of women, majority of white race (86%), 24% breast cancer, 57% GI cancer, and 22% pancreatic cancer. There was a similar proportion of Stages 3 and 4 cancer. Advanced stage matched comparison patients had an average of 1.73 (SD = 1.54) comorbidities at the time of baseline. There were no statistically significant differences between the ENACT advanced stage patients and the concurrent comparisons.

### 3.1 All patients

An examination of healthcare utilization and associated costs in all ENACT participants and matched comparisons show that even after accounting for intervention costs and adjusting for the number of pre‐existing comorbidities, the overall costs were lower among the ENACT participants as compared to the match comparisons (Table 2). Although these differences were not statistically significant, it can be noted, descriptively, that ENACT patients had fewer instances of ED visits (0.42 ± 0.07) and days spent in the hospital (1.40 ± 0.37) compared to matched comparisons (ED visits: 0.52 ± 0.12; Hospitalization: 2.00 ± 0.61). Therefore, ENACT patients spent less on ED visits (Arithmetic: $581.64, Geometric: $506.00) and hospitalization stays (Arithmetic: $14 980.00, Geometric: $6915.00) compared to the matched comparisons for both ED visits (Arithmetic: $22.28, Geometric: $583.00) and hospitalizations (Arithmetic: $21 400.00, Geometric: $6011.00). The total cost of the exercise intervention and healthcare utilization were higher in the matched comparison group ($22 122.28 ± 6614.82) compared to ENACT patients ($15 753.32 ± 3963.25). The geometric mean of total costs for ENACT patients was also lower at $3366.00 compared to the matched comparison group at $3600.00 (95% CI: −4571, 3959). As the number of comorbidities increased, the difference in healthcare utilization costs increased between all ENACT patients and matched comparisons. Among patients with no comorbidities (*n* = 83, 35.32%), ENACT patients spent $3149.00 (95% CI: −64, 7125) compared to $5439.00 (95% CI: −6750, 11 330) in all matched comparisons (MD = $2290.00, 95% CI: −6750, 11 330, *p* = .62). By comparison, among patients with three or more comorbidities (*n* = 46, 19.57%), ENACT patients spent $2187.00 (95% CI: −729, 5103) versus $16 003.00 (95% CI: −3159, 35 166) in matched comparisons (MD = $13 816.00, 95% CI: −5566, 33 199, *p* = .16). There were no statistically significant differences between groups.

**TABLE 2:** Healthcare utilization and costs—All participants

Cost component	Unit cost	ENACT participants (*N* = 160)			Matched comparisons (*N* = 75)			Comparison value and (95% confidence bounds)^a^
		Mean # of visit days (SE)	Arithmetic mean of total costs (Mean [SE])	Geometric mean of total costs (Mean [95% CI])	Mean # of visit days (SE)	Arithmetic mean of total costs (Mean [SE])	Geometric mean of total costs (Mean [95% CI])	
Healthcare costs								
ED visits	$1389	0.42 (0.07)	$581.64 (94.98)	$506 (306, 705)	0.52 (0.12)	$722.28 (175.62)	$583 (413, 753)	$110 (−165, 386)
Hospitalization (total nights)	$10 700	1.40 (0.37)	$14 980.00 (3923.41)	$6915 (4046, 9783)	2.00 (0.61)	$21 400.00 (6512.52)	$6011 (2897, 9125)	$−743 (−6806, 5320)
Intervention costs								
Screening	$1/person	1	$191.68	N/A	0	$0.00	N/A	N/A
Staff time for sessions, including wages and fringe	$28.77/h	6.1			0			
Equipment	$15/person	1			0			
Total costs								
Payers perspective			$15 753.32 (3963.25)	$3366 (1305, 5427)		$22 122.28 (6614.82)	$3600 (789, 6410)	$306 (−4571, 3959)

^a^ Comparison based on mixed method analysis that accounts for leading zeros and adjusts for number of comorbidities.

### 3.2 Advanced stage patients

In Table 3, we re‐examined healthcare utilization and associated costs in patients with Stages 3 and 4 breast, GI, and pancreatic cancer. After accounting for intervention costs and adjusting for the number of pre‐existing comorbidities, the overall costs were lower among the ENACT participants as compared to the match comparisons. However, there were no statistically significant differences in healthcare costs between groups. Although these differences were not statistically significant, descriptively, advanced stage patients in ENACT had fewer instances of ED visits (0.48 ± 0.11) and days spent in the hospital (1.01 ± 0.38) compared to matched comparisons (ED visits: 0.65 ± 0.22; Hospitalization: 2.51 ± 1.00). Thus, advanced stage ENACT patients spent less on ED visits (Arithmetic: $665.15, Geometric: $562.00) and hospitalization stays (Arithmetic: $10 850.70, Geometric: $6136.00) compared to matched comparisons for both ED visits (Arithmetic: $900.37, Geometric: $658.00) and hospitalization stays (Arithmetic: $26 894.59, Geometric: $10 142.00). The total cost at the payers' perspective was lower for advanced stage ENACT patients ($11 707.54 ± 4130.85) compared to matched comparisons ($27 795.57 ± 10 901.30). The geometric mean of total costs in advanced stage ENACT patients was also lower ($2817.00) compared to the matched comparison group ($6670.00) (95% CI: −3861, 9802). In advanced stage patients, a similar trend was seen for increased differences in healthcare utilization costs with a higher number of comorbidities between ENACT and matched comparison patients. Thirty‐four (31.48%) of advanced stage patients presented with no comorbidities. Total healthcare utilization costs for ENACT patients with no comorbidities were $6847.00 (95% CI: −3405, 17 099) compared to $8117.00 (95% CI: −3353, 19 586) in all matched comparisons (MD = $1270.00, 95% CI: −14 139, 16 679, *p* = .87). By contrast, among patients with three or more comorbidities (*n* = 21, 19.44%), ENACT patients spent $2170.00 (95% CI: −1081, 5420) versus $40 792.00 (95% CI: −16 998, 98 583) in matched comparisons (MD = $38 622.00, 95% CI: −19 259, 96 504, *p* = .19). There were no statistically significant differences between groups.

**TABLE 3:** Healthcare utilization and costs—Advanced stage breast, GI and pancreatic patients

Cost component	Unit cost	ENACT participants (*N* = 71)			Matched comparisons (*N* = 37)			Comparison value and (95% confidence bounds)^a^
		Mean # of visit days (SE)	Arithmetic mean of total costs (Mean [SE])	Geometric mean of total costs (Mean [95% CI])	Mean # of visit days (SE)	Arithmetic mean of total costs (Mean [SE])	Geometric mean of total costs (Mean [95% CI])	
Healthcare costs								
ED Visits	$1389	0.48 (0.11)	$665.15 (152.26)	$562 (411, 713)	0.65 (0.22)	$900.37 (310.20)	$658 (362, 953)	$67 (−270, 404)
Hospitalization (total nights)	$10 700	1.01 (0.38)	$10 850.70 (4081.04)	$6136 (2508, 9764)	2.51 (1.00)	$26 894.59 (10648.64)	$10 142 (4246, 16 038)	3656 (−4496, 11 808)
Intervention costs								
Screening	$1/person	1	$191.68	N/A	0	0	N/A	N/A
Staff time for sessions, including wages and fringe	$28.77/h	6.1			0			
Equipment	$15/person	1			0			
Total costs								
Payers perspective			$11 707.54 (4130.85)	$2817 (758, 4876)		$27 795.57 (10901.30)	$6670 (736, 12 604)	$2970 (−3861, 9802)

^a^ Comparison based on mixed method analysis that accounts for leading zeros and adjusts for number of comorbidities.

## 4 DISCUSSION

This study demonstrates that for patients who underwent chemotherapy, participation in the ENACT exercise oncology program was associated with numerically fewer ED visits and hospitalizations. These comparisons were made after including the cost of the intervention. Therefore, at the very least, the exercise oncology intervention may be cost neutral.

Our sub‐analysis of advanced stage breast, GI, and pancreatic cancer showed similar results. Advanced stage ENACT participants also visited the ED less and stayed in the hospital an average of 0.91 days less than matched comparisons. After accounting for the cost of the intervention, the average total direct costs in advanced stage breast, GI, and pancreatic cancer was $16 088.03 less in patients who participated in the exercise intervention compared to matched comparison patients who did not participate in the exercise intervention. Our findings in advanced stage breast, GI, and pancreatic cancer patients are especially important given the increased number of patients with three or more comorbidities and the trend of increasing healthcare utilization and associated costs with advancing stages of cancer. Although there was no statistically significant difference in total costs in advanced stage patients, there was a positive trend of increased costs in matched comparison patients with three or more comorbidities versus ENACT participants with three or more comorbidities (*p* = .18). Further, our cohort had a higher number of baseline comorbidities compared to the standard population.^21^ This suggests exercise during treatment for advanced stage patients may play a strong role in mitigating healthcare utilization and associated costs.

Investigations into treatment costs for women with breast cancer found that treatment costs in Stage 3, and 4 were 95%, and 109% higher than Stage 1.^22^ Although the observed cost differences did not reach statistical significance, a post‐hoc power analysis suggests that a future study with a sample size of 150 advanced cancer patients would yield 80% statistical power for detecting statistical significance for the observed difference between ENACT participants and matched comparisons, setting significance at an alpha of 0.05. Given that nearly 50% of the recruited patients in ENACT were advanced stage cancers, potential cost savings could be detected in future exercise oncology programs and be highly beneficial to advanced stage cancers.

Prior studies that have examined the cost effectiveness of exercise oncology programming have largely been conducted outside of the United States including the UK, Netherlands, and Australia. These countries have government‐based single‐payer healthcare structures compared to the fee‐for‐service private insurance environment of the United States. These large differences in organization of care and payment structures make it difficult to apply the findings to the U.S. healthcare environment.^23^, ^24^, ^25^, ^26^, ^27^, ^28^, ^29^ Specifically, costs for hospitalization stays and ED admissions vary between country due to different reimbursement and coverage models with the United States consistently having higher hospital expenditures compared to other high‐income countries.^30^ Countries such as the Netherlands and Australia provide coverage for exercise interventions, which is not applicable in the United States. To our knowledge, ours is the first study to evaluate cost effectiveness of exercise among patients currently undergoing active therapy.

The cost of the ENACT intervention was only $191.68 per patient. For the cost of this intervention to be matched by the cost of reduced hospitalizations, the number of hospitalization days that would have to be prevented over 500 patients is 0.02 days per patient (or a total of 8.96 total hospital days). Our preliminary data clearly demonstrates that this is possible to achieve, given the observation of 0.5 fewer hospital days per patient (see Table 2). Our prior publication noted that the exercise intervention was highly acceptable and feasible from the perspective of clinicians and patients alike,^16^ suggesting potential for future larger cost‐effectiveness trials.

Because the ENACT trial was designed to be pragmatic, the analysis conducted was appropriate. However, we acknowledge our analysis had some limitations. ENACT was not a randomized controlled trial, thus for this report, we conducted a post hoc secondary analysis of data from a single group pragmatic trial. Despite this, we were successful in identifying matched comparisons for ENACT trial participants (Table 1). There was one variable which the matched comparisons differed (comorbidities) and the analysis was adjusted for this difference. We also recognize this cost analysis does not include all direct healthcare costs, direct non‐healthcare costs, indirect costs, and travel costs, therefore, it cannot be viewed from the societal perspective.

Despite international guidelines promoting the implementation of exercise in cancer treatment from the American College of Sports Medicine,^9^, ^10^ little is known about the effects of exercise oncology interventions on healthcare utilization and healthcare costs. Understanding effects on healthcare costs would facilitate incorporating exercise as a standard and reimbursable form of treatment. Future research is needed to conduct a more thorough cost‐effectiveness analysis that would include indirect costs and provide a more detailed and holistic picture of the cost effectiveness of exercise during treatment.

## 5 CONCLUSION

Exercise is an effective strategy for symptom control with several international guidelines suggesting exercise should be implemented into standard of care during chemotherapy. However, only 23% of cancer patients and survivors are being referred to exercise by oncology physicians and only 30%–47% of cancer survivors are participating in exercise. A primary major barrier to referral and implementation to exercise oncology programs is the perceived burden of cost. This preliminary analysis suggests that an exercise oncology program is at least cost neutral, thus alleviating the financial burden of patients, payers, and society. Larger, well‐powered trials are required to determine whether there may be cost savings.

## CONFLICT OF INTEREST

The authors declare no conflict of interest.

## AUTHOR CONTRIBUTIONS

All authors had full access to the data in the study and take responsibility for the integrity of the data and the accuracy of the data analysis. *Conceptualization*, M.P., S.E.D., V.M.C., H.W., L.W., R.L., K.H.S.; *Investigation*, M.P., S.E.D., V.M.C., H.W., L.W., R.L., K.H.S; *Formal Analysis*, M.P., V.M.C., H.W., K.H.S; *Writing‐Original Draft*, M.P., S.E.D., V.M.C., H.W., L.W., R.L., K.H.S; *Writing‐Review & Editing*, M.P., S.E.D., V.M.C., H.W., L.W., R.L., K.H.S; *Visualization*, M.P., S.E.D., V.M.C., H.W., L.W., R.L., K.H.S; *Supervision*, M.P., K.H.S.; *Project Administration*, M.P., S.E.D., K.H.S.; *Funding Acquisition*, K.H.S.

## ETHICAL STATEMENT

This study was in accordance with the ethical standards of the institutional review board at the Penn State College of Medicine and with the 1964 Helsinki declaration and its later amendments or comparable ethical standards. Informed consent was obtained from all individual participants included in the study.

## Data Availability

Data sharing is not applicable to this article as no new data were created or analyzed in this study.
